# Inequalities on an extended Bessel function

**DOI:** 10.1186/s13660-018-1656-4

**Published:** 2018-03-27

**Authors:** Rosihan M. Ali, See Keong Lee, Saiful R. Mondal

**Affiliations:** 1 School of Mathematical Sciences, Universiti Sains Malaysia, USM Penang, Malaysia; 2 Department of Mathematics and Statistics, College of Science, King Faisal University, Al-Hasa, Saudi Arabia

**Keywords:** 33C10, 26D7, 26D15, Generalized Bessel function, Bessel function, Turán-type inequality, Monotonicity properties, Log-convexity

## Abstract

This paper studies an extended Bessel function of the form

$$ {}_{a}\mathtt{B}_{b, p, c}(x):= \sum _{k=0}^{\infty }\frac{(-c)^{k}}{k! \Gamma { ( a k +p+\frac{b+1}{2} ) } } \biggl( \frac{x}{2} \biggr) ^{2k+p}. $$

Representation formulations for ${}_{a}\mathtt{B}_{b,p, c}$ are derived in terms of the parameters *a*, *b*, and *p*. An important consequence is the derivation of an $(a+1)$-order differential equation satisfied by the function ${}_{a}\mathtt{B}_{b,p, c}$. Interesting functional inequalities are established, particularly for the case $a=2$, and $c=\pm \alpha^{2}$.

Monotonicity properties of ${}_{a}\mathtt{B}_{b,p, c}$ are also studied for non-positive *c*. Log-concavity and log-convexity properties in terms of the parameters *d* and *p* are respectively investigated for the closely related function

$$ {}_{a}\mathcal{B}^{d}_{b,p, c}(x): =\sum _{k=0}^{\infty }\frac{(-c/4)^{k} \Gamma { ( p+\frac{b+1}{2} ) }}{ \Gamma{ ( k+1 ) } \Gamma { ( ak+p+\frac{b+1}{2} ) }} \frac{(d)_{k}}{k!}x^{k}, $$

which leads to direct and reverse Turán-type inequalities.

## Introduction

The Bessel function of the first kind of order *p* given by

$$ J_{p}(x):= \sum_{k=0}^{\infty } \frac{(-1)^{k}}{k! \Gamma { ( k+p+1 ) } } \biggl( \frac{x}{2} \biggr) ^{2k+p}, \quad x \in\mathbb{R}, $$

is a particular solution of the homogeneous Bessel differential equation

$$\begin{aligned} x^{2} y''(x)+ x y'(x)+ \bigl(x^{2}-p^{2} \bigr)y(x)= 0. \end{aligned}$$

Here Γ denotes the gamma function. A solution of the homogeneous modified Bessel equation

$$\begin{aligned} x^{2} y''(x)+ x y'(x)- \bigl(x^{2}+p^{2} \bigr)y(x)= 0 \end{aligned}$$

gives the modified Bessel function of order *p*

$$ I_{p}(x) = \sum_{k=0}^{\infty } \frac{1}{k! \Gamma { ( k +p+1 ) } } \biggl( \frac{x}{2} \biggr) ^{2k+p}. $$

Because of its importance, the Bessel function and other special functions are of continued interest to the wider scientific community. The Bessel function and its variations have gone through several generalizations, see, for example, [1–6]. These generalized functions have also been framed as complex-valued analytic functions in the unit disk. Geometric properties of such functions have been studied, notably in [7–13].

Among the several generalized forms, perhaps a more complete generalization is that given by Baricz in [1]. In this case, the generalized Bessel function takes the form

1 $$\begin{aligned} {}_{a}\mathtt{B}_{b, p, c}(x):= \sum _{k=0}^{\infty }\frac{(-c)^{k}}{k! \Gamma { ( a k +p+\frac{b+1}{2} ) } } \biggl( \frac{x}{2} \biggr) ^{2k+p} \end{aligned}$$

for $a \in \mathbb{N}=\{1, 2, 3, \ldots \}$, and $b, p, c, x \in \mathbb{R}$. It is evident that the function ${}_{a}\mathtt{B}_{b, p, c}$ converges absolutely at each $x \in \mathbb{R}$. Earlier, Galué [14] introduced a generalization of the Bessel function of the form

$$ {}_{a}J_{p}(x):= \sum_{k=0}^{\infty } \frac{(-1)^{k}}{k! \Gamma {(a k +p+1)}} \biggl( \frac{x}{2} \biggr) ^{2k+p}, \quad x \in \mathbb{R}, a \in \mathbb{N}. $$

Apparently not much has been investigated for the extended Bessel function given by (1). Presumably such extensions would readily follow from recent results along similar used arguments, albeit involving intense computations. Still several pertinent questions remain, which include the question on how the parameter *a* influences the shape of the differential equation satisfied by ${}_{a}\mathtt{B}_{b, p, c}$. It is the aim of this paper to complement and to fill the void of earlier investigations on the Bessel function and its extensions.

The connection between the parameters *a*, *b*, and *p* in the representation formulae and recurrence relation for ${}_{a}\mathtt{B} _{b,p, c}$ are derived in Section 2. An important consequence is the derivation of an $(a+1)$-order differential equation satisfied by the function ${}_{a}\mathtt{B}_{b,p, c}$. As applications, new functional inequalities for ${}_{a}\mathtt{B}_{b, p, -\alpha^{2}}$ are obtained, particularly in the case $a=2$.

Section 3 is devoted to the investigation of the monotonicity properties of ${}_{a}\mathtt{B}_{b,p, c}$ for non-positive *c*, as well as for the normalized generalized Bessel function. Log-concavity and log-convexity properties in terms of the parameters *d* and *p* are also respectively investigated for the closely related function

$$ {}_{a}\mathcal{B}^{d}_{b,p, c}(x): = \sum_{k=0}^{\infty }\frac{(-c/4)^{k} \Gamma { ( p+\frac{b+1}{2} ) }}{ \Gamma { ( k+1 ) } \Gamma { ( ak+p+ \frac{b+1}{2} ) }} \frac{(d)_{k}}{k!}x^{k}. $$

As a consequence, direct and reverse Turán-type inequalities [15] are obtained.

## General representation formulations and applications

This section aims to find representation formulations, including integral representations, for the generalized function ${}_{a} \mathtt{B}_{b, p, c}$ in terms of the parameters *a*, *b*, and *p*. A starting point is the Gauss multiplication theorem [16] for the gamma function, which states that

$$\begin{aligned} \Gamma (mz)=(2\pi )^{\frac{1-m}{2}}m^{mz-\frac{1}{2}} \prod _{j=1}^{m} \Gamma \biggl( z+ \frac{j-1}{m} \biggr) , \quad z \neq 0,- \frac{1}{m},\ldots , \end{aligned}$$

$m \in \mathbb{N}$. Thus

2 $$\begin{aligned} \Gamma (ak+l) &= \Gamma \biggl( a \biggl( k+\frac{l}{a} \biggr) \biggr) \\ &=(2\pi )^{\frac{1-a}{2}}a ^{ak+l-\frac{1}{2}} \prod_{j=1}^{a}\Gamma \biggl( k+ \frac{l+j-1}{a} \biggr) \\ &=(2\pi )^{\frac{1-a}{2}}a^{ak+l-\frac{1}{2}}\prod_{j=1}^{a} \biggl( \frac{l+j-1}{a} \biggr) _{k} \Gamma \biggl( \frac{l+j-1}{a} \biggr) , \end{aligned}$$

$l \neq -ak,-ak-1, -ak-2,\ldots $ , and $k \in \mathbb{N}$. Here $(\alpha )_{k}$ is the Pochhammer symbol defined by $(\alpha )_{k}= \alpha (\alpha +1)_{k-1}= \Gamma (\alpha +k)/ \Gamma (\alpha )$, with $(\alpha )_{0}=1$. Substituting $z= l/a$ and $m=a$ gives

$$\begin{aligned} \prod_{j=1}^{a} \Gamma \biggl( \frac{l+j-1}{a} \biggr) =\frac{\Gamma (l)}{(2\pi )^{\frac{1-a}{2}}a^{l-\frac{1}{2}}}, \end{aligned}$$

and thus (2) yields

$$\begin{aligned} \Gamma (ak+l) =a^{ak} \Gamma {(l)}\;\prod _{j=1} ^{a} \biggl( \frac{l+j-1}{a} \biggr) _{k}. \end{aligned}$$

Choosing $l=p+(b+1)/2$, it is evident from (1) that

$$ {}_{a}\mathtt{B}_{b, p, c}(x)= \frac{x^{p}}{2^{p} \Gamma { ( p+\frac{b+1}{2} ) }}\sum _{k=0}^{\infty }\frac{1}{ ( \frac{2p+b+1}{2a} ) _{k} ( \frac{2p+b+3}{2a} ) _{k}\cdots ( \frac{2p+b+2a-1}{2a} ) _{k} (1)_{k}} \biggl( - \frac{c x^{2}}{4 a^{a}} \biggr) ^{k}, $$

which leads to the following representation in terms of the generalized hypergeometric function (see [17]):

$$\begin{aligned} {}_{m} F_{n}(a_{1},a_{2}, \ldots , a_{m}; b_{1},b_{2}, \ldots , b_{n};x)= \sum_{k=0}^{\infty }\frac{ (a_{1})_{k} (a_{2})_{k} \cdots (a _{m})_{k}}{ (b_{1})_{k} (b_{2})_{k} \cdots (b_{n})_{k} k!} x^{k}. \end{aligned}$$

### Proposition 1

*Let* $a \in \mathbb{N}$, *and* $b, p, c, x \in \mathbb{R}$. *Then*

$$\begin{aligned} {}_{a}\mathtt{B}_{b, p, c}(x)=\frac{x^{p}}{2^{p} \Gamma { ( p+\frac{b+1}{2} ) }} {}_{0} F_{a} \biggl( -; \frac{2p+b+1}{2a}, \frac{2p+b+3}{2a} ,\ldots , \frac{2p+b+2a-1}{2a};-\frac{c x^{2}}{4 a^{a}} \biggr) . \end{aligned}$$

Another representation formula can be expressed in terms of the order $a=1$. In the sequel, we shall simply write $\mathtt{B}_{b, p, c} := {}_{1}\mathtt{B}_{b, p, c}$. Thus

3 $$ \mathtt{B}_{b, p, c}(x):= \sum_{k=0}^{\infty } \frac{(-c)^{k}}{k! \Gamma { ( k +p+\frac{b+1}{2} ) }} \biggl( \frac{x}{2} \biggr) ^{2k+p}. $$

### Proposition 2

*Let* $a \in \mathbb{N}$, *and* $b, p, c, x \in \mathbb{R}$. *Then*

$$ {}_{a} \mathtt{B}_{b, p, c}(x)=(2 \pi )^{\frac{a-1}{2}}a^{-p- \frac{b}{2}} \biggl( \frac{x}{2} \biggr) ^{p} \prod _{j=1}^{a} \biggl( \frac{x}{2 a^{a/2}} \biggr) ^{-\frac{p+j-1}{a}} \mathtt{B}_{\frac{b+1-a}{a}, \frac{p+j-1}{a}, c} \biggl( \frac{x}{ a^{a/2}} \biggr) , $$

*where* $\mathtt{B}_{b, p, c}$ *is given by* (3).

### Proof

It is clear from (2) that

4 $$\begin{aligned} \Gamma \biggl( a k+p+\frac{b+1}{2} \biggr) = (2 \pi )^{\frac{1-a}{2}} a ^{ak+p+\frac{b}{2}} \prod_{j=1}^{a} \Gamma \biggl( k+\frac{p+ \frac{b+1}{2}+j-1}{a} \biggr) . \end{aligned}$$

Thus,

$$\begin{aligned} {}_{a} \mathtt{B}_{b, p, c}(x) &= \sum _{k=0}^{\infty }\frac{(-c)^{k}}{(2 \pi )^{\frac{1-a}{2}} a^{ak+p+\frac{b}{2}} k!\; \prod_{j=1}^{a} \Gamma ( k+\frac{p+\frac{b-1}{2}+j}{a} ) } \biggl( \frac{x}{2} \biggr) ^{2k+p} \\ &=(2 \pi )^{\frac{a-1}{2}}a^{\frac{-2p-b}{2}} \biggl( \frac{x}{2} \biggr) ^{p} \prod_{j=1}^{a} \biggl( \frac{x}{2 a^{a/2}} \biggr) ^{- \frac{p+j-1}{a}} \\ & \quad {} \times \sum_{k=0}^{\infty } \frac{(-c)^{k}}{ k!\; \Gamma ( k+ \frac{p+j-1}{a}+\frac{b+1}{2a} ) } \biggl( \frac{x}{2 a^{a/2}} \biggr) ^{2k+\frac{p+j-1}{a}} \\ &=(2 \pi )^{\frac{a-1}{2}}a^{\frac{-2p-b}{2}} \biggl( \frac{x}{2} \biggr) ^{p} \prod_{j=1}^{a} \biggl( \frac{x}{2 a^{a/2}} \biggr) ^{- \frac{p+j-1}{a}}\mathtt{B}_{\frac{b+1-a}{a}, \frac{p+j-1}{a}, c} \biggl( \frac{x}{ a^{a/2}} \biggr) . \end{aligned}$$

 □

### Remark 1

As a first application, let $a=2$. In this case, Proposition 2 yields

$$\begin{aligned} {}_{2} \mathtt{B}_{b, p, c}(x) =\sqrt{\frac{ \pi }{x 2^{b-3}}} \mathtt{B}_{\frac{b-1}{2}, \frac{p}{2}, c} \biggl( \frac{x}{ 2} \biggr) \mathtt{B}_{\frac{b-1}{2},\frac{p+1}{2}, c} \biggl( \frac{x}{ 2} \biggr) . \end{aligned}$$

Now $\mathtt{B}_{1,p, 1}(x)= J_{p}(x)$ is the classical Bessel function, while $\mathtt{B}_{1,p,-1}(x)= I_{p}(x)$ is the modified Bessel function of the first kind of order *p*. Thus, for $b=3$ and $c=\pm 1$, it follows that

$$\begin{aligned} {}_{2} \mathtt{B}_{3, p, 1}(x) &=\sqrt{\frac{ \pi }{x}}\; J_{ \frac{p}{2}} \biggl( \frac{x}{ 2} \biggr) J_{\frac{p+1}{2}} \biggl( \frac{x}{ 2} \biggr) \end{aligned}$$

and

$$\begin{aligned} {}_{2} \mathtt{B}_{3, p, -1}(x) &=\sqrt{\frac{ \pi }{x}}\; I_{ \frac{p}{2}} \biggl( \frac{x}{ 2} \biggr) I_{\frac{p+1}{2}} \biggl( \frac{x}{ 2} \biggr) . \end{aligned}$$

Thus interestingly

$$ \frac{{}_{2} \mathtt{B}_{3, 2p, 1}(x)}{{}_{2} \mathtt{B}_{3,2p+1, 1}(x)} = \frac{J_{p}(x/2)}{J_{p+1}(x/2)} \quad \text{and} \quad \frac{{} _{2} \mathtt{B}_{3, 2p, -1}(x)}{{}_{2} \mathtt{B}_{3,2p+1, -1}(x)} = \frac{I _{p}(x/2)}{I_{p+1}(x/2)}. $$

For obtaining recurrence relations, first differentiate (1) to yield

$$\begin{aligned} \frac{d}{dx} \bigl( x^{-p}{ }_{a} \mathtt{B}_{b, p, c}(x) \bigr) =& \sum_{k=0}^{\infty } \frac{(-c) ^{k}(k)}{2^{p} \Gamma ( ak+p+ \frac{b+1}{2} ) \Gamma ( k+1 ) }(x/2)^{2k-1} \\ =&\sum_{k=0}^{\infty }\frac{(-c) ^{k+1}(k+1)}{2^{p} \Gamma ( ak+p+\frac{b+1}{2} ) \Gamma ( k+2 ) } (x/2)^{2k+1} \\ =&-c x^{1-a-p} \biggl( \frac{1}{2} \biggr) ^{1-a}\sum _{k=0}^{\infty }\frac{(-c)^{k} }{\Gamma ( ak+p+\frac{b+1}{2} ) \Gamma ( k+ 1 ) } \biggl( \frac{x}{2} \biggr) ^{2k+p+a} \\ =&-c x^{-p} \biggl( \frac{x}{2} \biggr) ^{1-a}{ }_{a}\mathtt{B}_{b, p+a, c}(x). \end{aligned}$$

Expanding the left side of the above equation yields

5 $$ x \frac{d}{dx}{}_{a}\mathtt{B}_{b, p,c}(x) = p { }_{a}\mathtt{B}_{b, p, c}(x) -c \biggl( \frac{x}{2} \biggr) ^{1-a} x { }_{a}\mathtt{B}_{b, p+a, c}(x). $$

Yet another form for $x{}_{a}\mathtt{B}_{b, p,c}'$ is obtained from

$$\begin{aligned} \frac{d}{dx} \bigl( x_{{}}^{\frac{2p+b-1}{a}-p}{ }_{a} \mathtt{B}_{b, p, c}(x) \bigr) &= \sum_{k=0}^{\infty } \frac{(-c)^{k} }{2^{2k+p}\Gamma ( ak+p+\frac{b+1}{2} ) \Gamma ( k+1 ) }\frac{d}{dx}x^{2k+\frac{2p+b-1}{a}} \\ &= \sum_{k=0}^{\infty } \frac{(-c)^{k} (\frac{2}{a}) }{2^{2k+p}\Gamma ( ak+p+\frac{b+1}{2} ) \Gamma ( k+1 ) }x^{2k+\frac{2p+b-1-a}{a}} \\ &=\frac{1}{a}\;x^{\frac{2p+b-1}{a}-p}\text{ } {}_{a}B_{b, p-1,c} ( x ) . \end{aligned}$$

Expanding the left side of the above relation, it follows that

6 $$\begin{aligned} x \frac{d}{dx}{}_{a}\mathtt{B}_{b, p,c}(x) =\frac{x}{a}{}_{a} \mathtt{B}_{a, p-1,c}(x) - \biggl( \frac{2p+b-1}{a}- p \biggr) {}_{a} \mathtt{B}_{b, p,c}(x). \end{aligned}$$

Thus (5) and (6) lead to the following recurrence relation.

### Proposition 3

*Let* $a \in \mathbb{N}$, *and* $b, p, c, x \in \mathbb{R}$. *Then*

$$ \frac{x}{a}{}_{a}\mathtt{B}_{b, p-1,c}(x)+ c \biggl( \frac{x}{2} \biggr) ^{1-a} x { }_{a} \mathtt{B}_{b, p+a, c}(x)= \biggl( \frac{2p+b-1}{a} \biggr) {}_{a}\mathtt{B}_{b, p,c}(x). $$

We next find an $(a+1)$-order differential equation satisfied by ${ }_{a}\mathtt{B}_{b, p, c}$ from the recurrence relations (5) and (6) (see also [18]).

### Theorem 1

*Let the operator* *D* *be given by* $D := x (d/dx)$. *For each* $k=1, \ldots , a$, *the generalized Bessel function* ${}_{a}\mathtt{B}_{b, p,c}$ *satisfies the differential equation*

7 $$\begin{aligned} (D-p)\prod_{j=1}^{k} \biggl( D+\frac{2p+b+1-2j}{a}-p \biggr) {}_{a} \mathtt{B}_{b, p,c}(x) &+\frac{cx^{k+2-a}}{a^{k} 2^{1-a}} {}_{a} \mathtt{B}_{b, p-k+a,c}(x) = 0. \end{aligned}$$

*In particular*, *the generalized Bessel function* ${}_{a}\mathtt{B}_{b, p,c}$ *is a solution of the differential equation*

8 $$ (D-p)\prod_{j=1}^{a} \biggl( D+\frac{2p+b+1-2j}{a}-p \biggr) y(x)+\frac{cx ^{2}}{a^{a} 2^{1-a}} y(x)= 0. $$

### Proof

The proof is by induction. In terms of the differential operator *D*, identity (6) takes the form

9 $$\begin{aligned} \biggl( D+\frac{2p+b-1}{a}- p \biggr) {}_{a} \mathtt{B}_{b, p,c}(x) = \frac{x}{a}{}_{a} \mathtt{B}_{b, p-1,c}(x) . \end{aligned}$$

Now identity (5) gives

$$\begin{aligned} D \bigl(x {}_{a}\mathtt{B}_{b, p-1,c}(x) \bigr) &= x^{2} {}_{a}\mathtt{B}'_{b, p-1,c}(x)+ x {}_{a} \mathtt{B}_{b, p-1,c}(x) \\ &=p x{ }_{a}\mathtt{B}_{b, p-1, c}(x)-c \biggl( \frac{x}{2} \biggr) ^{1-a} x^{2} { }_{a} \mathtt{B}_{b, p-1+a, c}(x) \\ &=p a \biggl( D+\frac{2p+b-1}{a}- p \biggr) {}_{a} \mathtt{B}_{b, p,c}(x) -c \biggl( \frac{x}{2} \biggr) ^{1-a} x^{2} { }_{a}\mathtt{B}_{b, p-1+a, c}(x). \end{aligned}$$

Applying the operator *D* to both sides of (9), the latter equation leads to

$$\begin{aligned} &D \biggl( D+\frac{2p+b-1}{a}- p \biggr) {}_{a} \mathtt{B}_{b, p,c}(x) \\ &\quad = \frac{1}{a} D \bigl(x{}_{a} \mathtt{B}_{b, p-1,c}(x) \bigr) \\ &\quad =p \biggl( D+\frac{2p+b-1}{a}- p \biggr) {}_{a} \mathtt{B}_{b, p,c}(x) -\frac{c}{a 2^{1-a}} x^{3-a} { }_{a}\mathtt{B}_{b, p-1+a, c}(x), \end{aligned}$$

whence

$$\begin{aligned} &(D-p) \biggl( D+\frac{2p+b-1}{a}- p \biggr) {}_{a} \mathtt{B}_{b, p,c}(x) + \frac{c}{a 2^{1-a}} x^{3-a} { }_{a}\mathtt{B}_{b, p-1+a, c}(x)=0. \end{aligned}$$

This establishes (7) for $k=1$.

Assume now that (7) holds for $k=n$. It follows from (6) that

$$\begin{aligned} &D \bigl(x^{n-a+2} { }_{a}\mathtt{B}_{b, p-n+a, c}(x) \bigr) \\ &\quad = x^{n-a+3} { }_{a} \mathtt{B}'_{b, p-n+a, c}(x)+(n-a+2)x^{n-a+2}{ }_{a}\mathtt{B}_{b, p-n+a, c}(x) \\ &\quad = \frac{x^{n+3-a}}{a} { }_{a}\mathtt{B}_{b, p-n-1+a, c}(x)- \biggl( \frac{2(p-n)+b-1}{a}-p \biggr) x^{n-a+2}{ }_{a}\mathtt{B} _{b, p-n+a, c}(x). \end{aligned}$$

Applying the operator *D* to both sides of (7) for $k=n$, the above equation shows that

$$\begin{aligned} &D(D-p)\prod_{j=1}^{n} \biggl( D+ \frac{2p+b+1-2j}{a}-p \biggr) {}_{a} \mathtt{B}_{b, p,c}(x) \\ &\quad =-\frac{c}{a^{n+1} 2^{1-a}} x^{n+3-a} { }_{a}\mathtt{B}_{b, p-n-1+a, c}(x) \\ & \quad \quad {}+ \frac{c}{a^{n} 2^{1-a}} \biggl( \frac{2(p-n)+b-1}{a}-p \biggr) x^{n-a+2} { }_{a}\mathtt{B}_{b, p-n+a, c}(x). \end{aligned}$$

The induction formula allows us to rewrite the final term above in the form

$$\begin{aligned} &D(D-p)\prod_{j=1}^{n} \biggl( D+ \frac{2p+b+1-2j}{a}-p \biggr) {}_{a} \mathtt{B}_{b, p,c}(x) \\ &\quad =-\frac{c}{a^{n+1} 2^{1-a}} x^{n+3-a} { }_{a}\mathtt{B}_{b, p-n-1+a, c}(x) \\ &\quad \quad {}- \biggl( \frac{2(p-n)+b-1}{a}-p \biggr) \Biggl[ (D-p)\prod _{j=1} ^{n} \biggl( D+\frac{2p+b+1-2j}{a}-p \biggr) {}_{a}\mathtt{B}_{b, p,c}(x) \Biggr] . \end{aligned}$$

Thus

$$\begin{aligned} & \biggl( D+\frac{2(p-n)+b-1}{a}-p \biggr) (D-p)\prod _{j=1}^{n} \biggl( D+ \frac{2p+b+1-2j}{a}-p \biggr) {}_{a}\mathtt{B}_{b, p,c}(x) \\ &\quad =-\frac{c}{a^{n+1} 2^{1-a}} x^{n+3-a} { }_{a} \mathtt{B}_{b, p-n-1+a, c}(x), \end{aligned}$$

that is,

$$\begin{aligned} (D-p)\prod_{j=1}^{n+1} \biggl( D+ \frac{2p+b+1-2j}{a}-p \biggr) {}_{a} \mathtt{B}_{b, p,c}(x) &+ \frac{cx^{n+3-a}}{a^{n+1} 2^{1-a}} {}_{a} \mathtt{B}_{b, p-n-1+a,c}(x) =0. \end{aligned}$$

 □

### Remark 2

For $a=1$, the differential equation (8) reduces to

$$ x^{2} y''(x) + bxy'(x) + \bigl(c x^{2}-p^{2} +(1-b)p \bigr) y(x)=0. $$

This is the differential equation considered by Baricz [2] in his study on the unification of Bessel, modified Bessel, spherical Bessel, and modified spherical Bessel functions. Thus the differential equation yields the Bessel function of the first kind of order *p* when $b=c=1$, and the modified Bessel function of the first kind of order *p* when $b=1$ and $c=-1$. In the case $b=2$ and $c=1$, there results the spherical Bessel function of order *p*.

For $a=2$, (8) reduces to

$$ x^{3} y'''(x)+(1+b-p) x^{2} y''(x)+(b-1) \biggl( \frac{b+1}{4}-p \biggr) x y'(x) + \biggl( \frac{c}{2}x^{2}-p \frac{(b-1)(b-3)}{4} \biggr) y(x)=0. $$

Thus its particular solution is ${}_{2}\mathtt{B}_{b, p, c}$, which from Proposition 1 can be expressed in the form

$$\begin{aligned} {}_{2}\mathtt{B}_{b, p, c}(x) &= \sum _{k=0}^{\infty }\frac{(-c)^{k}}{k! \Gamma { ( 2 k +p+\frac{b+1}{2} ) } } \biggl( \frac{x}{2} \biggr) ^{2k+p} \\ &=\frac{x^{p}}{2^{p} \Gamma {(p+\frac{b+1}{2})}} {}_{0} F_{2} \biggl( -; \frac{2p+b+1}{4}, \frac{2p+b+3}{4};- \frac{c x^{2}}{16} \biggr) . \end{aligned}$$

We conclude this section by establishing two integral representations for ${}_{a}\mathtt{B}_{b, p, c}$. For this purpose, first recall the integral form of the beta function $B(x,y)$ [16, 17] given by

10 $$\begin{aligned} B(x,y):=\frac{ \Gamma {(x)} \Gamma {(y)}}{\Gamma {(x+y)}}= \int_{0}^{1} t^{x-1}(1-t)^{y-1}\,dt \end{aligned}$$

for $\operatorname{Re}x >0, \operatorname{Re}y >0$. Replacing *x* by $(a k+1)$ and *y* by $(2 p+b-1)/2$ in (10), we get

$$\begin{aligned} \frac{1}{ \Gamma ( a k+ p +\frac{b+1}{2} ) }= \frac{2}{\Gamma {(a k+1)} \Gamma { ( \frac{2p+b-1}{2} ) }} \int _{0}^{1} t^{2a k+1} \bigl(1-t^{2} \bigr)^{\frac{2p+b-3}{2}}\,dt, \end{aligned}$$

where $p> -(b-1)/2$.

For $a \in \mathbb{N}$, identity (2) yields

$$ \Gamma { ( ak+1 ) }= \Gamma { \biggl( a \biggl( k+ \frac{1}{a} \biggr) \biggr) }=(2\pi )^{\frac{1-a}{2}}a^{ak+ \frac{1}{2}} \prod_{j=1}^{a} \Gamma \biggl( k+ \frac{j}{a} \biggr) . $$

Then the generalized Bessel function ${}_{a}\mathtt{B}_{b, p, c}$ takes the form

$$\begin{aligned} {}_{a}\mathtt{B}_{b, p, c}(x) &= \frac{2 ( \frac{x}{2} ) ^{p}}{a ^{1/2}(2\pi )^{(1-a)/2} \Gamma { ( \frac{2p+b-1}{2} ) }} \prod_{j=1}^{a} \int_{0}^{1} t \bigl(1-t^{2} \bigr)^{\frac{2p+b-3}{2}} \sum_{k=0}^{\infty } \frac{(-c)^{k} }{k! \Gamma ( k+\frac{j}{a} ) } \biggl( \frac{x t^{a}}{2 a^{a/2}} \biggr) ^{2k}\,dt \\ &=\frac{2 ( \frac{x}{2} ) ^{p}}{a^{1/2}(2\pi )^{(1-a)/2} \Gamma { ( \frac{2p+b-1}{2} ) }} \prod_{j=1}^{a} \biggl( \frac{x }{2 a^{a/2}} \biggr) ^{1-\frac{j}{a}} \int_{0}^{1} t^{1-a+j} \bigl(1-t^{2} \bigr)^{ \frac{2p+b-3}{2}} \\ & \quad {} \times \sum_{k=0}^{\infty } \frac{(-c)^{k} }{k! \Gamma ( k+\frac{j}{a}-1+1 ) } \biggl( \frac{x t^{a}}{2 a^{a/2}} \biggr) ^{2k+\frac{j}{a}-1}\,dt \\ &=\frac{2 ( \frac{x}{2} ) ^{p}}{a^{1/2}(2\pi )^{(1-a)/2} \Gamma { ( \frac{2p+b-1}{2} ) }} \\ & \quad {} \times \prod_{j=1}^{a} \biggl( \frac{x }{2 a^{a/2}} \biggr) ^{1- \frac{j}{a}} \int_{0}^{1} t^{1-a+j} \bigl(1-t^{2} \bigr)^{\frac{2p+b-3}{2}} \mathtt{B}_{1, (j/a)-1, c} \biggl( \frac{x t^{a}}{a^{a/2}} \biggr)\,dt, \end{aligned}$$

which establishes the following proposition.

### Proposition 4

*Let* $a \in \mathbb{N}$, *and* $b, p, c, x \in \mathbb{R}$. *Then*

$$\begin{aligned} {}_{a}\mathtt{B}_{b, p, c}(x)&=\frac{2^{1-p}x^{p}}{a^{1/2}(2\pi )^{(1-a)/2} \Gamma { ( p+\frac{b-1}{2} ) }} \\ &\quad {}\times\prod _{j=1}^{a} \biggl( \frac{x }{2 a ^{a/2}} \biggr) ^{1-\frac{j}{a}} \int_{0}^{1} t^{1-a+j} \bigl(1-t^{2} \bigr)^{ \frac{2p+b-3}{2}} \mathtt{B}_{1, (j/a)-1, c} \biggl( \frac{x t^{a}}{a ^{a/2}} \biggr)\,dt. \end{aligned}$$

### Remark 3

The particular cases $a=b=1=\pm c$ in Proposition 4 respectively lead to

$$\begin{aligned}& {}_{1}\mathtt{B}_{1, p, 1}(x)=J_{p}(x) = \frac{2 ( \frac{x}{2} ) ^{p}}{\Gamma (p)} \int_{0}^{1} t \bigl( 1-t^{2} \bigr) ^{p-1} J_{0}(x t)\,dt, \\& {}_{1}\mathtt{B}_{1, p, -1}(x)=I_{p}(x) = \frac{2 ( \frac{x}{2} ) ^{p}}{\Gamma (p)} \int_{0}^{1} t \bigl( 1-t^{2} \bigr) ^{p-1} I_{0}(x t)\,dt, \end{aligned}$$

for $x>0$ and $p>0$.

Another integral representation is the following.

### Proposition 5

*Let* $a \in \mathbb{N}$, *and* $b, p, c, x \in \mathbb{R}$. *Then*

$$\begin{aligned} {}_{a}\mathtt{B}_{b, p, c}(x) & =\frac{2^{1-p} x^{p}}{\sqrt{\pi } (2 \pi )^{{\frac{1-a}{2}}}a^{p+\frac{b}{2}}} \prod _{j=1}^{a}\frac{1}{\Gamma { ( \frac{2p+2j+b-1-a}{2a} ) }} \\ & \quad {} \times \int_{0}^{1}\sum_{k=0}^{\infty } \frac{(-c)^{k}}{(2k)!} \biggl( \frac{xt}{ a^{a/2}} \biggr) ^{2k} \bigl(1-t^{2} \bigr)^{\frac{2p+2j+b-1-3a}{2a}}\,dt. \end{aligned}$$

### Proof

Replace *x* by $(k+1/2)$ and *y* by $((2p+2j+b-1-a)/2a)$ in (10). Then

11 $$ \frac{1}{ \Gamma ( k+\frac{2 p+2 j+b-1}{2 a} ) }= \frac{2}{\Gamma { ( k+\frac{1}{2} ) } \Gamma { ( \frac{2p+2j+b-1-a}{2a} ) }} \int_{0}^{1} t^{2k} \bigl(1-t^{2} \bigr)^{\frac{2p+2j+b-1-3a}{2a}}\,dt, $$

where $p>(a-1-b)/2$. On the other hand, (4) yields

12 $$\begin{aligned} \Gamma { \biggl( ak+p+\frac{b+1}{2} \biggr) } &= \Gamma { \biggl( a \biggl( k+\frac{2p+b+1}{2a} \biggr) \biggr) } \\ &=(2\pi )^{\frac{1-a}{2}}a^{ak+\frac{2p+b}{2}}\prod_{j=1}^{a} \Gamma \biggl( k+\frac{2p+b+2j-1}{2a} \biggr) . \end{aligned}$$

Thus (11) and (12) shows that the generalized Bessel function ${}_{a}\mathtt{B}_{b, p, c}$ takes the form

13 $$\begin{aligned} {}_{a}\mathtt{B}_{b, p, c}(x) &=\sum _{k=0}^{\infty }(-c)^{k} \frac{ ( \frac{x}{2} ) ^{2k+p}}{\Gamma {(k+1)} \Gamma ( k+ \frac{1}{2} ) }\frac{2}{(2\pi )^{\frac{1-a}{2}}a^{ak+p+ \frac{b}{2}}} \\ & \quad {} \times \prod_{j=1}^{a} \frac{1}{ \Gamma { ( \frac{2p+2j+b-1-a}{2a} ) }} \int _{0}^{1} t^{2k} \bigl(1-t^{2} \bigr)^{\frac{2p+2j+b-1-3a}{2a}}\,dt \\ & =\frac{2 ( \frac{x}{2} ) ^{p}}{(2\pi )^{{\frac{1-a}{2}}}a ^{p+\frac{b}{2}}} \prod_{j=1}^{a} \frac{1}{ \Gamma { ( \frac{2p+2j+b-1-a}{2a} ) }} \int _{0}^{1}\sum_{k=0}^{\infty } \frac{(-c)^{k}(xt)^{2k}(1-t^{2})^{ \frac{2p+2j+b-1-3a}{2a}}}{\Gamma ( k+1 ) \Gamma ( k+ \frac{1}{2} ) (2 a^{a/2})^{2k}}\,dt. \end{aligned}$$

Now the Legendre duplication formula (see [16, 17])

$$ \Gamma {(z)} \Gamma { \biggl( z+ \frac{1}{2} \biggr) }= 2^{1-2z} \; \sqrt{\pi } \Gamma {(2z)} $$

shows that

$$\begin{aligned} \Gamma {(k+1)}\; \Gamma { \biggl( k+\frac{1}{2} \biggr) }=k \Gamma {(k)} \Gamma { \biggl( k+\frac{1}{2} \biggr) }= 2^{1-2k} k\sqrt{ \pi } \Gamma {(2k)} = \frac{\sqrt{\pi } (2k)!}{2^{2k}}. \end{aligned}$$

This reduces (13) to the desired representation and completes the proof. □

### Remark 4

For another application, choose $c=\pm \alpha^{2}$. Then Proposition 5 leads to

$$\begin{aligned} {}_{a}\mathtt{B}_{b, p, \alpha^{2}}(x) & = \frac{2^{1-p}x^{p}}{\sqrt{ \pi } (2\pi )^{{\frac{1-a}{2}}}a^{p+\frac{b}{2}}} \prod_{j=1}^{a} \frac{1}{\Gamma { ( \frac{2p+2j+b-1-a}{2a} ) }} \\ & \quad {} \times \int_{0}^{1}\sum_{k=0}^{\infty } \frac{(-1)^{k}}{(2k)!} \biggl( \frac{ \alpha xt}{ a^{a/2}} \biggr) ^{2k} \bigl(1-t^{2} \bigr)^{\frac{2p+2j+b-1-3a}{2a}}\,dt \\ & =\frac{{2^{1-p}x^{p}}}{\sqrt{\pi } (2\pi )^{{\frac{1-a}{2}}}a ^{p+\frac{b}{2}}} \prod_{j=1}^{a} \frac{1}{ \Gamma { ( \frac{2p+2j+b-1-a}{2a} ) }} \int _{0}^{1} \bigl(1-t^{2} \bigr)^{\frac{2p+2j+b-1-3a}{2a}} \cos \biggl( \frac{\alpha xt}{ a^{a/2}} \biggr)\,dt, \end{aligned}$$

and

$$\begin{aligned} {}_{a}\mathtt{B}_{b, p, -\alpha^{2}}(x) & =\frac{{2^{1-p}x^{p}}}{\sqrt{ \pi } (2\pi )^{{\frac{1-a}{2}}}a^{p+\frac{b}{2}}} \prod _{j=1}^{a}\frac{1}{\Gamma { ( \frac{2p+2j+b-1-a}{2a} ) }} \\ & \quad {} \times \int_{0}^{1}\sum_{k=0}^{\infty } \frac{1}{(2k)!} \biggl( \frac{ \alpha xt}{ a^{a/2}} \biggr) ^{2k} \bigl(1-t^{2} \bigr)^{\frac{2p+2j+b-1-3a}{2a}}\,dt \\ & =\frac{{2^{1-p}x^{p}}}{\sqrt{\pi } (2\pi )^{{\frac{1-a}{2}}}a ^{p+\frac{b}{2}}} \prod_{j=1}^{a} \frac{1}{ \Gamma { ( \frac{2p+2j+b-1-a}{2a} ) }} \int _{0}^{1} \bigl(1-t^{2} \bigr)^{\frac{2p+2j+b-1-3a}{2a}} \cosh \biggl( \frac{\alpha xt}{ a^{a/2}} \biggr)\,dt. \end{aligned}$$

Substituting $t=\cos \theta $ yields

$$\begin{aligned} {}_{a}\mathtt{B}_{b, p, \alpha^{2}}(x) &= \frac{-2^{1-p}x^{p}}{\sqrt{ \pi } (2\pi )^{{\frac{1-a}{2}}}a^{\frac{2p+b}{2}}} \prod_{j=1}^{a}\frac{1}{\Gamma { ( \frac{2p+2j+b-1-a}{2a} ) }} \\ & \quad \times \int_{\frac{\pi }{2}}^{0} \bigl(1-\cos^{2} \theta \bigr)^{ \frac{2p+2j+b-1-3a}{2a}} \cos \biggl( \frac{\alpha x \cos \theta }{ a ^{\frac{a}{2}}} \biggr) \sin \theta \,d\theta \\ &=\frac{2^{1-p}x^{p}}{\sqrt{\pi } (2\pi )^{{\frac{1-a}{2}}}a^{ \frac{2p+b}{2}}} \prod_{j=1}^{a} \frac{1}{ \Gamma { ( \frac{2p+2j+b-1-a}{2a} ) }} \\ & \quad {}\times \int_{0}^{\frac{\pi }{2}} (\sin \theta )^{ \frac{2p+2j+b-1-3a}{a}+1} \cos \biggl( \frac{\alpha x \cos (\theta )}{ a ^{\frac{a}{2}}} \biggr)\,d\theta \\ & =\frac{2^{1-p}x^{p}}{\sqrt{\pi } (2\pi )^{{\frac{1-a}{2}}}a^{ \frac{2p+b}{2}}} \prod_{j=1}^{a} \frac{1}{ \Gamma { ( \frac{2p+2j+b-1-a}{2a} ) }} \\ &\quad {}\times \int _{0}^{\frac{\pi }{2}} (\sin \theta )^{\frac{2p+2j+b-1-2a}{a}} \cos \biggl( \frac{\alpha x \cos \theta }{ a^{\frac{a}{2}}} \biggr)\,d\theta , \end{aligned}$$

and

$$\begin{aligned} {}_{a}\mathtt{B}_{b, p, -\alpha^{2}}(x) & =\frac{2^{1-p}x^{p}}{\sqrt{ \pi } (2\pi )^{{\frac{1-a}{2}}}a^{\frac{2p+b}{2}}} \prod _{j=1}^{a}\frac{1}{\Gamma { ( \frac{2p+2j+b-1-a}{2a} ) }} \\ &\quad {}\times \int_{0}^{ \frac{\pi }{2}} (\sin \theta )^{\frac{2p+2j+b-1-2a}{a}} \cosh \biggl( \frac{ \alpha x \cos \theta }{ a^{\frac{a}{2}}} \biggr)\,d\theta . \end{aligned}$$

The particular case $a=b=1=\alpha $ in the above representations gives respectively the integral representation for the classical Bessel and modified Bessel functions of order *p*:

$$\begin{aligned} {}_{1}\mathtt{B}_{1, p, 1}(x)=J_{p}(x) &= \frac{2^{1-p}x^{p}}{\sqrt{ \pi } \Gamma { ( p +\frac{1}{2} ) }} \int_{0}^{\frac{ \pi }{2}} (\sin \theta )^{2p} \cos ( x \cos \theta )\,d\theta \\ &=\frac{ ( \frac{x}{2} ) ^{p}}{\sqrt{\pi } \Gamma { ( p +\frac{1}{2} ) }} \int_{0}^{\pi } (\sin \theta )^{2p} \cos ( x \cos \theta )\,d\theta \\ &=\frac{2^{1-p}x^{p}}{\sqrt{\pi } \Gamma { ( p + \frac{1}{2} ) }} \int_{0}^{1} \bigl(1-t^{2} \bigr)^{p-\frac{1}{2}} \cos ( x t )\,dt, \end{aligned}$$

and

$$\begin{aligned} {}_{1}\mathtt{B}_{1, p, -1}(x)=I_{p}(x) & = \frac{ ( \frac{x}{2} ) ^{p}}{\sqrt{\pi } \Gamma { ( p +\frac{1}{2} ) }} \int_{0}^{\pi } (\sin \theta )^{2p} \cosh ( x \cos \theta )\,d\theta \\ & =\frac{2^{1-p}x^{p}}{\sqrt{\pi } \Gamma { ( p + \frac{1}{2} ) }} \int_{0}^{1} \bigl(1-t^{2} \bigr)^{p-\frac{1}{2}} \cosh ( x t )\,dt. \end{aligned}$$

These integrations for $J_{p}$ and $I_{p}$ can also be found in [16, 9.1.20, p. 360] and [16, 9.6.18, p. 376].

## Monotonicity and consequences

Investigations into the monotonicity properties of the generalized function ${}_{a}\mathtt{B}_{b,p,c}$ hinges on the following result of Biernacki and Krzyż [19].

### Lemma 1

([19])

*Suppose* $f(x)=\sum_{k=0}^{\infty }a_{k} x^{k}$ *and* $g(x)=\sum_{k=0}^{\infty }b_{k} x^{k}$, *where* $a_{k} \in \mathbb{R}$ *and* $b_{k} > 0$ *for all k*. *Further suppose that both series converge on* $\vert x \vert < r$. *If the sequence* $\{a_{k}/b_{k}\}_{k\geq 0}$ *is increasing* (*or decreasing*), *then the function* $x \mapsto f(x)/g(x)$ *is also increasing* (*or decreasing*) *on* $(0,r)$.

Evidently, the above lemma also holds true when both *f* and *g* are even functions, or both odd.

### Theorem 2

*Let* $c \leq 0$.

- *If* $q \geq p > -(b+1)/2$ *and* $a \leq d$, *then* $x \mapsto ( 2^{p}x^{-p}{}_{a}\mathtt{B}_{b,p,c}(x) ) / ( 2^{q}x^{-q} {}_{d}\mathtt{B}_{b,q,c}(x) ) $ *is increasing on* $(0, \infty )$.
- *The function* $p\mapsto {}_{a}\mathtt{B}_{b,p+a,c}(x)/{} _{a}\mathtt{B}_{b,p,c}(x)$ *is decreasing on* $(-(b+1)/2, \infty )$ *for each fixed* $x>0$.
- *The function* $x\mapsto x{}_{a}\mathtt{B}_{b,p,c}'(x)/{} _{a}\mathtt{B}_{b,p,c}(x)$ *is increasing on* $(0, \infty )$ *for each fixed* $p > -{(b+1)}/{2}$.

### Proof

(a) From (1) it is evident that

$$\begin{aligned} \frac{ x^{q-p}{}_{a}\mathtt{B}_{b,p,c}(x)}{2^{q-p}{}_{b}B_{b,q,c}(x)}=\frac{ \sum_{k=0}^{\infty }\alpha_{k, p,a} ( \frac{x}{2} ) ^{2k}}{ \sum_{k=0}^{\infty }\alpha_{k, q,d} ( \frac{x}{2} ) ^{2k}}, \end{aligned}$$

where

$$ \alpha_{k, p,a}=\frac{(-c)^{k}}{k! \Gamma { ( ak+p+ \frac{b+1}{2} ) }} \quad \text{and} \quad \alpha_{k, q,d}=\frac{(-c)^{k}}{k! \Gamma { ( dk+q+\frac{b+1}{2} ) }}. $$

Write $w_{k}= \alpha_{k,p,a}/\alpha_{k,q,d}$; since $d \geq a$ and $q \geq p$, it follows that

$$\begin{aligned} \frac{w_{k+1}}{w_{k}} &=\frac{ \Gamma { ( ak+p+ \frac{b+1}{2} ) } \Gamma { ( dk+d+q+\frac{b+1}{2} ) }}{ \Gamma { ( dk+q+\frac{b+1}{2} ) } \Gamma { ( ak+a+p+\frac{b+1}{2} ) }}=\frac{ ( d k +q+\frac{b+1}{2} ) _{d}}{ ( a k+p+\frac{b+1}{2} ) _{a}} \geq 1. \end{aligned}$$

The result now readily follows from Lemma 1.

(b) Let $q \geq p>-(b+1)/2$. It follows from part (a) that

$$\begin{aligned} \frac{d}{dx} \biggl( \frac{2^{p} x^{-p}{}_{a}\mathtt{B}_{b,p,c}(x)}{ {2^{q} x^{-q}{}_{a}\mathtt{B}_{b,q,c}(x)}} \biggr) \geq 0 \end{aligned}$$

on $(0,\infty )$. Thus

$$\begin{aligned} \bigl( x^{-p}{}_{a}\mathtt{B}_{b,p,c}(x) \bigr) ' \bigl( x^{-q}{}_{a} \mathtt{B}_{b,q,c}(x) \bigr) - \bigl( x^{-p}{}_{a} \mathtt{B}_{b,p,c}(x) \bigr) \bigl( x^{-q}{}_{a} \mathtt{B}_{b,q,c}(x) \bigr) '\geq 0. \end{aligned}$$

It now follows from (5) that

$$\begin{aligned} (-c)\;x^{-p-q} \biggl( \frac{x}{2} \biggr) ^{1-a} \bigl( {}_{a}\mathtt{B} _{b,p+a,c}(x)\; {}_{a} \mathtt{B}_{b,q,c}(x)-{}_{a}\mathtt{B}_{b,p,c}(x) \;{}_{a}\mathtt{B}_{b,q+a,c}(x) \bigr) \geq 0, \end{aligned}$$

whence ${}_{a} B_{b,p+a,c}/{}_{a} B_{b,p,c}$ is decreasing for $p>-(b+1)/2$.

(c) Let $\beta_{k,p,a}:=(2k+p)\alpha_{k, p,a}$. Then the quotient $x {}_{a}\mathtt{B}_{b,p,c}'/{}_{a}\mathtt{B}_{b,p,c}$ can be written as

$$\begin{aligned} \frac{x {}_{a}B'_{b,p,c}(x)}{{}_{a}\mathtt{B}_{b,p,c}(x)}= \frac{ \sum_{k=0}^{\infty }{\beta_{k,p,a} ( \frac{x}{2} ) ^{2k}}}{ \sum_{k=0}^{\infty }{\alpha_{k,p,a} ( \frac{x}{2} ) ^{2k}}}. \end{aligned}$$

Clearly, the sequence $\{\beta_{k,p,a}/\alpha_{k,p,a}\}_{k \geq 0}=\{2k+p \}_{k\geq 0}$ is increasing, and hence Lemma 1 shows that the function $x \mapsto x {}_{a}\mathtt{B}_{b,p,c}'/{}_{a}\mathtt{B} _{b,p,c}$ is increasing on $( 0, \infty ) $. □

Next consider the normalized function

14 $$\begin{aligned} {}_{a}\mathcal{B}_{b,p,c}(x)&=2^{p}x^{-p} \Gamma { \biggl( p+\frac{b+1}{2} \biggr) } {}_{a}\mathtt{B}_{b, p,c}(x) \\ &= \Gamma { \biggl( p+\frac{b+1}{2} \biggr) }\sum_{k=0}^{\infty }\frac{(-c)^{k}}{k! \Gamma { ( ak+p+\frac{b+1}{2} ) }} \biggl( \frac{x}{2} \biggr) ^{2k}. \end{aligned}$$

Also let ${}_{1}\Phi_{1}$ be the confluent hypergeometric function

$$ {}_{1}\Phi_{1}(\alpha ; \beta ; x)= \sum _{k=0}^{\infty }\frac{ ( \alpha )_{k} x^{k}}{(\beta )_{k} k!}. $$

The next result discusses the monotonicity property of rational functions involving ${}_{a}\mathcal{B}_{b,p,c}$.

### Theorem 3

*Let* $c \leq 0$.

- *If* $\alpha \geq \beta >0$, *then the function* $x \mapsto {}_{a}\mathcal{B}_{b,p,c}(x)/{}_{1}\Phi_{1}(\alpha ; \beta ; -c x^{2}/4)$ *is decreasing on* $\mathbb{R}$ *for each fixed* $p > -{(b+1)}/ {2}$.
- *If* $0< \beta \leq (2p+b+1)/(2a)$, *then the function* $x \mapsto {}_{a}\mathcal{B}_{b,p,c}(x)/F_{a}(\beta ; -c x^{2}/4)$ *is decreasing on* $\mathbb{R}$ *for each fixed* $p > -{(b+1)}/{2}$, *where*
  $$ F_{a}(\beta , x):= {}_{0}F_{a} \biggl( -; \beta , \beta +\frac{1}{a}, \ldots , \beta +\frac{a-1}{a}; x \biggr) . $$

### Proof

(a) It follows from (14) that

$$ \frac{{}_{a}\mathcal{B}_{b, p, c}(x)}{{}_{1}\Phi_{1} ( \alpha ; \beta ; -c\frac{x^{2}}{4} ) }=\frac{\sum_{k=0}^{\infty }\delta _{1}(k) ( \frac{x}{2} ) ^{2k}}{\sum_{k=0}^{\infty }\delta _{2}(k) ( \frac{x}{2} ) ^{2k}} $$

with

$$ \delta_{1}(k)=\frac{(-c)^{k}\Gamma ( p+\frac{b+1}{2} ) }{k! \Gamma ( ak+p+\frac{b+1}{2} ) } \quad \text{and} \quad \delta _{2}(k)=\frac{(-c)^{k} (\alpha )_{k}}{k! (\beta )_{k}}. $$

Set $w_{k}:= \delta_{1}(k)/\delta_{2}(k)$. Since $\alpha \geq \beta $,

$$ \frac{w_{k+1}}{w_{k}}= \frac{(\beta +k)\Gamma ( ak+p+ \frac{b+1}{2} ) }{(\alpha +k)\Gamma ( ak+a+p+\frac{b+1}{2} ) } \leq 1. $$

Thus $\{w_{k}\}_{k}$ is decreasing, and the result follows from Lemma 1.

(b) From Proposition 1, a representation of ${}_{a} \mathcal{B}_{b, p, c}$ by the generalized hypergeometric function is

$$ {}_{a}\mathcal{B}_{b, p, c}(x)=\sum _{k=0}^{\infty }\sigma_{1}(k) \biggl( \frac{x}{2} \biggr) ^{2k}, $$

where

$$ \sigma_{1}(k):=\frac{(-c)^{k} }{a^{ak}k! \prod_{j=1}^{a} ( \frac{2p+b+2j-1}{2a} ) _{k}}. $$

Let

$$ \sigma_{2}(k):=\frac{(-c)^{k} }{k! \prod_{j=1}^{a} ( \beta + \frac{j-1}{a} ) _{k}}. $$

Then

$$ \frac{{}_{a}\mathcal{B}_{b, p, c}(x)}{F_{a} ( \beta ,-c\frac{x ^{2}}{4} ) } = \frac{\sum_{k=0}^{\infty }\sigma_{1}(k) ( \frac{x}{2} ) ^{2k}}{\sum_{k=0}^{\infty }\sigma_{2}(k) ( \frac{x}{2} ) ^{2k}}. $$

With $\tau_{k}:= \sigma_{1}(k)/\sigma_{2}(k)$, a computation shows that

$$ \frac{\tau_{k+1}}{\tau_{k}}= \frac{1}{a^{a}} \prod_{j=1}^{a} \frac{ ( \frac{2p+b+2j-1}{2a} ) _{k}}{ ( \frac{2p+b+2j-1}{2a} ) _{k+1}} \frac{ ( \beta +\frac{j-1}{a} ) _{k+1}}{ ( \beta + \frac{j-1}{a} ) _{k}} =\frac{1}{a^{a}} \prod _{j=1}^{a} \frac{ \beta +\frac{j-1}{a}+k}{\frac{2p+b+2j-1}{2a}+k}. $$

Now

$$ \frac{2p+b+2j-1}{2a} \geq \beta +\frac{j-1}{a} $$

for each fixed *j*, $1 \leq j \leq a$, provided $0 < \beta \leq (2p+b+1)/(2a)$. Hence $\tau_{k+1} \leq \tau_{k}$, and the result follows from Lemma 1. □

Another function of interest is that given by

15 $$ {}_{a}\mathcal{B}^{d}_{b,p, c}(x):= \sum_{k=0}^{\infty }\frac{(-c/4)^{k}\Gamma { ( p+\frac{b+1}{2} ) }}{ \Gamma { ( k+1 ) } \Gamma { ( ak+p+ \frac{b+1}{2} ) }} \frac{(d)_{k}}{k!}x^{k} =\sum_{k=0}^{\infty } \frac{(-c/4)^{k}(d)_{k}}{\Gamma{ ( k+1 ) } ( p+\frac{b+1}{2} ) _{ak} k!}x ^{k}. $$

Note that ${}_{a}\mathcal{B}^{1}_{b,p,c}(x)={}_{a}\mathcal{B}_{b,p,c}( \sqrt{x})$, where ${}_{a}\mathcal{B}_{b,p,c}$ is given by (14). The following result by Karp and Sitnik [20, Theorem 1] is required to deduce the log-concavity of ${}_{a}\mathcal{B}^{d}_{b,p, c}$ in terms of the parameter *d*.

### Lemma 2

([20])

*Let*

$$ f(d,x)=\sum_{k=0}^{\infty }f_{k} \frac{(d)_{k}}{k!} x^{k}, $$

*where* $f_{k}>0$ (*and is independent of* *d*). *Suppose* $e>d>0$, $\delta >0$. *Then the function*

$$ \phi_{d, e, \delta }(x)=f(d+\delta ,x)f(e,x)-f(e+\delta ,x)f(d,x)= \sum _{m=2}^{\infty }\phi_{m} x^{m} $$

*has positive power series coefficients* $\phi_{m} >0$ *so that* $d \mapsto f(d,x)$ *is strictly log*-*concave for* $x>0$ *whenever the sequence* $\{ f_{k}/f_{k-1} \} $ *is decreasing*. *On the other hand*, $\phi_{d, e, \delta }(x)$ *has negative power series coefficients* $\phi_{m} <0$ *so that* $d \mapsto f(d,x)$ *is strictly log*-*convex for* $x>0$ *whenever the sequence* $\{ f_{k}/f_{k-1} \} $ *is increasing*.

### Theorem 4

*Let* $c \leq 0$ *and* $d>0$.

- *The function*
  $$ p \mapsto {}_{a}\mathcal{B}^{d}_{b,p, c}(x) $$
  *given by* (15) *is decreasing and log*-*convex on* $(-(b+1)/2, \infty )$ *for each fixed* $x>0$ *and* $d>0$.
- *The function*
  $$ p \mapsto {}_{a}\mathcal{B}_{b,p+1, c}^{d}(x)/{}_{a} \mathcal{B}_{b,p, c}^{d}(x) $$
  *is increasing on* $(-(b+1)/2, \infty )$, *that is*, *for* $q \geq p >-(b+1)/2$, *the inequality*
  16 $$ {}_{a}\mathcal{B}_{b, q+1, c}^{d}(x){}_{a} \mathcal{B}_{b,p, c}^{d}(x) \geq {}_{a} \mathcal{B}_{b, q, c}^{d}(x){}_{a}\mathcal{B}_{b,p+1, c} ^{d}(x) $$
  *holds for each fixed* $x>0$ *and* $d>0$.
- *The function* $d \mapsto {}_{a}\mathcal{B}^{d}_{b,p, c}(x)$ *is log*-*concave on* $(0, \infty )$ *for each fixed* $x>0$ *and* $p >(2a-b-1)/2$.

### Proof

(a) Let $q\geq p > -(b+1)/2$. Then $(q+(b+1)/2)_{ak} > (p+(b+1)/2)_{ak}$ for all $k \in \{0, 1, 2, \ldots \}$. Thus

$$\begin{aligned} \gamma_{k}(q, d):=\frac{(-c)^{k}(d)_{k}}{4^{k} \Gamma { ( k+1 ) } ( q+\frac{b+1}{2} ) _{ak}k!} \leq \frac{(-c)^{k} (d)_{k}}{4^{k}\Gamma { ( k+1 ) } ( p+\frac{b+1}{2} ) _{ak}k!}:= \gamma_{k}(p, d). \end{aligned}$$

Since

$$ {}_{a}\mathcal{B}_{b,q, c}^{d}(x)=\sum _{k=0}^{\infty } \gamma_{k}(q, d) x^{k}, $$

we deduce that

$$\begin{aligned} {}_{a}\mathcal{B}_{b,q, c}^{d}(x) \leq {}_{a}\mathcal{B}_{b,p, c}^{d}(x) \end{aligned}$$

for each fixed $x >0$ and $d>0$. Therefore $p \mapsto {}_{a} \mathcal{B}_{b,p, c}^{d}$ is decreasing for $p > -(b+1)/2$.

To show log-convexity of ${}_{a}\mathcal{B}_{b,p, c}^{d}$, it suffices to show that $p \mapsto \gamma_{k}(p, d)$ is log-convex for all $k \in \{0,1, 2, 3, \ldots \}$ and fixed $d>0$. Then the result follows from the fact that sums of log-convex functions are also log-convex.

Let Ψ be the digamma function given by $\Psi (p)= \Gamma '{(p)}/ \Gamma {(p)}$. Then evidently

$$\begin{aligned} \frac{\partial^{2}}{\partial {p^{2}}} \bigl(\log \bigl(\gamma_{k}( p,d) \bigr) \bigr)= \Psi ' \biggl( p+\frac{b+1}{2} \biggr) -\Psi ' \biggl( ak+p+\frac{b+1}{2} \biggr) . \end{aligned}$$

Note that [16, p. 260] $\Psi '$ has the explicit form

$$\begin{aligned} \Psi '(t)=\sum_{n=0}^{\infty } \frac{1}{(t+n)^{2}}, \quad t \in \mathbb{R}\setminus \{0,-1,-2,\ldots \}. \end{aligned}$$

This implies that

$$\begin{aligned} \frac{\partial^{2}}{\partial {p^{2}}} \bigl(\log \bigl(\gamma_{k}(p, d) \bigr) \bigr)= \sum_{n=0}^{\infty }\frac{ak(ak+2p+(b+1)+2n)}{ ( p+\frac{b+1}{2}+n ) ^{2} ( ak+p+\frac{b+1}{2}+n ) ^{2}} \geq 0 \end{aligned}$$

for all $k \in \{0, 1, 2, \ldots \}$ and $p > -(b+1)/2$. Thus $p \mapsto \gamma_{k}( p, d)$ is log-convex on $(-(b+1)/2, \infty )$, and consequently ${}_{a}\mathcal{B}_{b,p, c}^{d}$ is log-convex for each fixed $x>0$.

(b) It is clear that (16) is equivalent to showing

$$\begin{aligned} \Biggl( \sum_{k=0}^{\infty } \gamma_{k}(q+1, d)x^{k} \Biggr) \Biggl( \sum _{k=0}^{\infty }\gamma_{k}(p, d)x^{k} \Biggr) \geq \Biggl( \sum_{k=0} ^{\infty } \gamma_{k}(q, d)x^{k} \Biggr) \Biggl( \sum _{k=0}^{\infty } \gamma_{k}(p+1, d)x^{k} \Biggr) , \end{aligned}$$

which holds whenever

17 $$\begin{aligned} \gamma_{i}(q+1, d)\gamma_{j}(p, d)+ \gamma_{j}(q+1, d) \gamma_{i}(p, d) \geq \gamma_{j}(q, d)\gamma_{i}(p+1, d)+\gamma_{i}(q, d) \gamma_{j}(p+1, d) \end{aligned}$$

for all $i, j \in \mathbb{N}$.

Let

$$ \lambda_{1}= \Gamma { \biggl( ai+q+\frac{b+3}{2} \biggr) } \Gamma { \biggl( aj+p+\frac{b+3}{2} \biggr) } $$

and

$$ \lambda_{2}= \Gamma { \biggl( aj+q+\frac{b+3}{2}\biggr) } \Gamma { \biggl( ai+p+\frac{b+3}{2} \biggr) }. $$

Then

18 $$\begin{aligned} &\gamma_{i}(q+1, d)\gamma_{j}(p, d)+\gamma_{j}(q+1, d) \gamma_{i}(p, d) \\ &\quad = \frac{(-c/4)^{i+j} \Gamma ( p+\frac{b+1}{2} ) \mathrm{ \Gamma } ( q+\frac{b+3}{2} ) (d)_{i} (d)_{j}}{ \Gamma ( i+1 ) \Gamma ( j+1 ) i! j!} \biggl[ \frac{aj+p+ \frac{b+1}{2}}{\lambda_{1}} +\frac{ai+q+\frac{b+1}{2}}{\lambda_{2}} \biggr] . \end{aligned}$$

Similarly,

19 $$\begin{aligned} &\gamma_{j}(q, d)\gamma_{i}(p+1, d)+\gamma_{i}(q, d) \gamma_{j}(p+1, d) \\ &\quad = \frac{ ( -c/4 ) ^{i+j} \Gamma ( q+ \frac{b+1}{2} ) \Gamma ( p+\frac{b+3}{2} ) (d)_{i} (d)_{j}}{ \Gamma ( i+1 ) \Gamma ( j+1 ) i! j!} \biggl[ \frac{ai+q+\frac{b+1}{2}}{ \lambda_{1}} +\frac{aj+p+\frac{b+1}{2}}{\lambda_{2}} \biggr] . \end{aligned}$$

Next, for $i \geq j$, relations (18) and (19) show that inequality (17) is equivalent to

$$\begin{aligned} & \biggl( q+\frac{b+1}{2} \biggr) \biggl[ \biggl( aj+p+\frac{b+1}{2} \biggr) \lambda_{2} + \biggl( ai+q+\frac{b+1}{2} \biggr) \lambda_{1} \biggr] \\ &\quad \geq \biggl( p+\frac{b+1}{2} \biggr) \biggl[ \biggl( aj+p+ \frac{b+1}{2} \biggr) \lambda_{1}+ \biggl( ai+q+\frac{b+1}{2} \biggr) \lambda_{2} \biggr] . \end{aligned}$$

Since $q\geq p$, this can be further simplified to showing

$$\begin{aligned} a ( \lambda_{1}-\lambda_{2} ) (i-j) \biggl( p+\frac{b+1}{2} \biggr) \geq 0. \end{aligned}$$

The latter inequality clearly holds true whenever $\lambda_{1} \geq \lambda_{2}$.

To see that this is indeed the case, for $q \geq p$, let

$$\begin{aligned} \phi (x):= \frac{ \Gamma { ( ax+ q +\frac{b+3}{2} ) }}{\mathrm{ \Gamma }{ ( ax+ p +\frac{b+3}{2} ) }}. \end{aligned}$$

Since $x \mapsto \Gamma {(ax+y)}$ is log-convex, it follows that $\phi '(x) \geq 0$. Thus $\phi (i) \geq \phi (j)$ for $i \geq j$, and consequently $\lambda_{1} \geq \lambda_{2}$. This validates inequality (16).

(c) To apply Lemma 2, let

$$ f_{k}:=\frac{(-c/4)^{k} {\Gamma ( p+\frac{b+1}{2} ) }}{ \Gamma (k+1) \Gamma ( ak+p+\frac{b+1}{2} ) }. $$

We shall show that the sequence $b_{k} = \{ f_{k}/f_{k-1} \} $ is decreasing. A calculation gives

$$ b_{k}= -\frac{c}{4} \frac{\Gamma ( ak+p+\frac{b+1}{2}-a ) }{k \; \Gamma ( ak+p+\frac{b+1}{2} ) }, $$

and so we need to show that the function $\xi :(0,\infty )\to \mathbb{R}$ given by

$$ \xi (x)= \frac{\Gamma ( ax+p+\frac{b+1}{2}-a ) }{x\; \Gamma ( ax+p+\frac{b+1}{2} ) } $$

is decreasing for $p>(2a-b-1)/2$. Logarithmic differentiation gives

$$\begin{aligned} \frac{\xi '(x)}{\xi (x)}= a \Psi \biggl( ax+p+\frac{b+1}{2}-a \biggr) - a \Psi \biggl( ax+p+\frac{b+1}{2} \biggr) -\frac{1}{x}. \end{aligned}$$

Since the digamma function is known to be increasing on $(0, \infty )$ for $p>(2a-b-1)/2$ and $x>0$, it follows that

$$\begin{aligned} \frac{\xi '(x)}{\xi (x)} < 0. \end{aligned}$$

Thus *ξ* is indeed decreasing, and Lemma 2 shows that the function

$$ d\mapsto {}_{a}\mathcal{B}_{b,p, c}^{d}(x) =\sum _{k=0}^{\infty } f _{k} \frac{(d)_{k}}{k!} x^{k} $$

is log-concave. □

The results of parts (a) and (b) in Theorem 4 in the case $d=1$ were also obtained by Baricz [1, Theorem 3, Theorem 4].

### Remark 5

Theorem 4 has interesting consequences, among which is the Tur$\acute{\text{a}}$n-type inequality for the function ${}_{a}\mathcal{B}_{b,p, c}^{d}$ given by (15). From the definition of log-convexity, it follows from Theorem 4(a) that

$$\begin{aligned} {}_{a}\mathcal{B}_{b, \alpha p_{1}+(1-\alpha )p_{2}, c}^{d}(x) \leq \bigl( {}_{a}\mathcal{B}_{b,p_{1}, c}^{d}(x) \bigr) ^{\alpha } \bigl( {} _{a}\mathcal{B}_{b,p_{2}, c}^{d}(x) \bigr) ^{1-\alpha }, \end{aligned}$$

where $\alpha \in [0,1]$, $p_{1}, p_{2} > -(b+1)/2$, and $x >0$. Choosing $\alpha =1/2$, $p_{1}=p-\nu $ and $p_{2}=p+\nu $, the above inequality yields

20 $$\begin{aligned} \bigl({}_{a}\mathcal{B}_{b,p,c}^{d}(x) \bigr)^{2} - {}_{a}\mathcal{B} _{b,p+\nu , c}^{d}(x) {}_{a}\mathcal{B}_{b,p-\nu }^{d}(x)\leq 0. \end{aligned}$$

On the other hand, the log-concavity of $d \mapsto {}_{a}\mathcal{B} _{b,p, c}^{d}$ implies that

$$\begin{aligned} {}_{a}\mathcal{B}_{b, p, c}^{td_{1}+(1-t)d_{2}}(x) \geq \bigl( {}_{a} \mathcal{B}_{b,p, c}^{d_{1}}(x) \bigr) ^{t} \bigl( {}_{a}\mathcal{B} _{b,p, c}^{d_{2}}(x) \bigr) ^{1-t} \end{aligned}$$

for $t \in [0, 1]$, $d_{2} > d_{1} >0$, $p> (2a-b-1)/2$ and $x>0$. Choosing $t=1/2$, $d_{1}=d-\mu $, and $d_{2}=d+\mu $, $\mu \in \mathbb{R}$, the inequality reduces to

21 $$\begin{aligned} \bigl( {}_{a}\mathcal{B}_{b, p, c}^{d}(x) \bigr) ^{2} \geq {}_{a} \mathcal{B}_{b,p, c}^{d+\mu }(x) {}_{a}\mathcal{B}_{b,p, c}^{d-\mu }(x). \end{aligned}$$

Thus (20) and (21) lead to direct and reverse Tur$\acute{\text{a}}$n-type inequalities

$$\begin{aligned} {}_{a}\mathcal{B}_{b,p, c}^{d+\mu }(x) {}_{a} \mathcal{B}_{b,p, c}^{d- \mu }(x)\leq \bigl( {}_{a} \mathcal{B}_{b, p, c}^{d}(x) \bigr) ^{2} \leq {}_{a}\mathcal{B}_{b,p+\nu , c}^{d}(x) {}_{a} \mathcal{B}_{b,p- \nu }^{d}(x). \end{aligned}$$

### Remark 6

For $d=2$, it follows from (15) that

22 $$\begin{aligned} {}_{a}\mathcal{B}^{2}_{b,p, c}(x) &=\sum_{k=0}^{\infty }\frac{(-c/4)^{k}\mathrm{ \Gamma }{ ( p+\frac{b+1}{2} ) }}{ \Gamma { ( k+1 ) } \Gamma { ( ak+p+ \frac{b+1}{2} ) }} \frac{(2)_{k}}{k!}x^{k} \\ &=\sum_{k=0}^{\infty }\frac{(-c/4)^{k} \Gamma { ( p+ \frac{b+1}{2} ) }}{ \Gamma { ( k+1 ) } \mathrm{ \Gamma }{ ( ak+p+\frac{b+1}{2} ) }} \frac{(k+1)!}{k!}x^{k} \\ &=x \sum_{k=1}^{\infty } \frac{(-c/4)^{k} \Gamma { ( p+ \frac{b+1}{2} ) }}{ \Gamma { ( k+1 ) } \mathrm{ \Gamma }{ ( ak+p+\frac{b+1}{2} ) }} kx^{k-1} \\ &\quad {}+ \sum_{k=0}^{ \infty } \frac{(-c/4)^{k} \Gamma { ( p+\frac{b+1}{2} ) }}{ \Gamma { ( k+1 ) } \Gamma { ( ak+p+\frac{b+1}{2} ) }} x^{k} \\ &= x \frac{d}{dx} \bigl( {}_{a}\mathcal{B}_{b,p, c} ( \sqrt{x} ) \bigr) + {}_{a}\mathcal{B}_{b,p, c} ( \sqrt{x} ) , \end{aligned}$$

where ${}_{a}\mathcal{B}_{b,p, c}$ is given by (14). With $d=1$ and $\mu =1$ in (21), then ${}_{a}\mathcal{B} _{b,p, c}^{1}(x)={}_{a}\mathcal{B}_{b,p, c} ( \sqrt{x} ) $. Thus (22) shows that

$$\begin{aligned} \bigl( {}_{a}\mathcal{B}_{b,p, c} ( \sqrt{x} ) \bigr) ^{2} \geq x \frac{d}{dx} \bigl( {}_{a} \mathcal{B}_{b,p, c} ( \sqrt{x} ) \bigr) + {}_{a} \mathcal{B}_{b,p, c} ( \sqrt{x} ) . \end{aligned}$$

### Remark 7

Inequality (16) leads to a generalization of the Turán-type inequality

$$ \bigl( {}_{a}\mathcal{B}_{b, p+1,c}^{d}(x) \bigr) ^{2}\leq {}_{a} \mathcal{B}_{b, p,c}^{d}(x) \;{}_{a}\mathcal{B}_{b, p+2,c}^{d}(x). $$

Inequality (16) also yields

$$\begin{aligned} \frac{{}_{a}\mathcal{B}_{b, q,c}^{d}(x)}{{}_{a}\mathcal{B}_{b,p,c} ^{d}(x)}\leq \frac{{}_{a}\mathcal{B}_{b, q+1,c}^{d}(x)}{{}_{a} \mathcal{B}_{b,p+1,c}^{d}(x)} \leq \frac{{}_{a}\mathcal{B}_{b, q+2,c} ^{d}(x)}{{}_{a}\mathcal{B}_{b,p+2,c}^{d}(x)}\leq \cdots \leq \frac{ {}_{a}\mathcal{B}_{b, q+a,c}^{d}(x)}{{}_{a}\mathcal{B}_{b,p+a,c}^{d}(x)}. \end{aligned}$$

Thus

$$ \frac{{}_{a}\mathcal{B}_{b, q,c}^{d}(x)}{{}_{a}\mathcal{B}_{b, p,c} ^{d}(x)}\leq \frac{{}_{a}\mathcal{B}_{b, q+a,c}^{d}(x)}{{}_{a} \mathcal{B}_{b, p+a,c}^{d}(x)}. $$

The next result gives a dominant function for ${}_{a}\mathtt{B}_{b,p, -\alpha^{2}}$.

### Theorem 5

*Let* $p\geq -(b+1)/2$ *and* $x \geq 0$. *Then*

$$ {}_{a}\mathtt{B}_{b,p, -\alpha^{2}}(x) \leq \frac{1}{\Gamma ( p+ \frac{b+1}{2} ) } \biggl( \frac{x}{2} \biggr) ^{p}\exp \biggl( \frac{ \alpha^{2} x^{2}}{4 ( p+\frac{b+1}{2} ) ^{a}} \biggr) . $$

### Proof

Clearly the estimate trivially holds for $x=0$. Let $x>0$. It is readily established by mathematical induction that $\Gamma (m+x) \geq x^{m} \Gamma (x)$ for $m \in \mathbb{N}$ and $x\geq 0$. Then

$$ \Gamma \biggl( a k + p+\frac{b+1}{2} \biggr) \geq \biggl( p+ \frac{b+1}{2} \biggr) ^{a k} \Gamma \biggl( p+\frac{b+1}{2} \biggr) , $$

and thus

$$\begin{aligned} {}_{a}\mathtt{B}_{b,p, -\alpha^{2}}(x) &\leq \frac{1}{\Gamma ( p+ \frac{b+1}{2} ) }\sum _{k=0}^{\infty }\frac{\alpha^{2k}}{ ( p+ \frac{b+1}{2} ) ^{ak} k!} \biggl( \frac{x}{2} \biggr) ^{2k+p} \\ &=\frac{1}{\Gamma ( p+\frac{b+1}{2} ) } \biggl( \frac{x}{2} \biggr) ^{p}\exp \biggl( \frac{\alpha^{2} x^{2}}{4 ( p+\frac{b+1}{2} ) } \biggr) ^{a} . \end{aligned}$$

 □

For $\alpha =\pm 1$, $b=a=1$, Theorem 5 leads to a dominant for the modified Bessel function

$$ I_{p}(x) \leq \frac{1}{\Gamma ( p+1 ) } \biggl( \frac{x}{2} \biggr) ^{p}e^{\frac{ x^{2}}{4 ( p+1 ) }} $$

obtained by Baricz in [21].

The final result uses the Chebyshev integral inequality [22, p. 40]: Suppose *f* and *g* are two integrable functions monotonic in the same sense (either both decreasing or both increasing). Let $q: (a, b) \to \mathbb{R}$ be a positive integrable function. Then

23 $$\begin{aligned} \biggl( \int_{a}^{b} q(t) f(t)\,dt \biggr) \biggl( \int_{a}^{b} q(t) g(t)\,dt \biggr) \leq \biggl( \int_{a}^{b} q(t)\,dt \biggr) \biggl( \int_{a}^{b} q(t) f(t) g(t)\,dt \biggr) . \end{aligned}$$

The inequality in (23) is reversed if *f* and *g* are monotonic of the opposite sense.

### Theorem 6

*Let* $p > -(b-1)/2$, $\alpha \in \mathbb{R}\backslash \{0\}$, *and* $x \in (0, \pi /\vert \alpha \vert )$. *Then*

$$\begin{aligned}& {}_{2}\mathtt{B}_{b, p, \alpha^{2}}(x) \geq \frac{ \pi \alpha^{1-p-\frac{b}{2}}x^{1-\frac{b}{2}} ( \Gamma ( \frac{2p+b}{4} ) ) ^{2}}{2^{\frac{5-b}{2}} \Gamma (\frac{2p+b-1}{4})\Gamma ( \frac{2p+b+1}{4})}\; \biggl( \mathtt{J}_{\frac{2p+b-2}{4}} \biggl( \frac{ \alpha x}{2} \biggr) \biggr) ^{2}, \\& {}_{2}\mathtt{B}_{b, p, -\alpha^{2}}(x) \geq \frac{ \pi \alpha^{1-p-\frac{b}{2}}x^{1-\frac{b}{2}} ( \Gamma ( \frac{2p+b}{4} ) ) ^{2}}{2^{\frac{5-b}{2}} \Gamma (\frac{2p+b-1}{4})\Gamma ( \frac{2p+b+1}{4})}\; \biggl( \mathtt{I}_{\frac{2p+b-2}{4}} \biggl( \frac{ \alpha x}{2} \biggr) \biggr) ^{2}. \end{aligned}$$

### Proof

Putting $a=2$ in Remark 4, the integral form for ${}_{2}\mathtt{B}_{b, p, \alpha^{2}}(x)$ is

$$\begin{aligned} {}_{2}\mathtt{B}_{b, p, \alpha^{2}}(x) & = \frac{x^{p}}{2^{2p+ \frac{b-3}{2}} { \Gamma { ( \frac{2p+b-1}{4} ) }} { \Gamma { ( \frac{2p+b+1}{4} ) }}} \biggl( \int_{0} ^{1} \bigl(1-t^{2} \bigr)^{\frac{2p+b-5}{4}} \cos \biggl( \frac{\alpha xt}{2} \biggr)\,dt \biggr) \\ & \quad {} \times \biggl( \int_{0}^{1} \bigl(1-t^{2} \bigr)^{\frac{2p+b-3}{4}} \cos \biggl( \frac{ \alpha xt}{2} \biggr)\,dt \biggr) . \end{aligned}$$

To establish the subordinant for ${}_{2}\mathtt{B}_{b, p, \alpha^{2}}$, let

$$ q(t) = \bigl(1-t^{2} \bigr)^{\frac{2p+b-3}{4}} \cos \biggl( \frac{\alpha x t}{2} \biggr) ; \quad \quad f(t)= g(t) := \bigl(1-t^{2} \bigr)^{-\frac{1}{4}}, \quad 0 < t < 1. $$

Then

$$\begin{aligned} \int_{0}^{1} q(t)f(t)\,dt= \int_{0}^{1} q(t)g(t)\,dt &= \int_{0}^{1} \bigl(1-t ^{2} \bigr)^{\frac{2p+b-4}{4}} \cos \biggl( \frac{\alpha x t}{2} \biggr)\,dt. \end{aligned}$$

It is known that for $\operatorname{Re} \nu >-1/2$, the classical Bessel function $\mathtt{J}_{\nu }$ has the integral representation

$$\begin{aligned} \mathtt{J}_{\nu }(y)= \frac{2^{1-\nu }y^{\nu }}{\sqrt{\pi } \mathrm{ \Gamma }{ ( \nu +\frac{1}{2} ) }} \int_{0}^{1} \bigl(1-t^{2} \bigr)^{ \nu -\frac{1}{2}} \cos ( y t )\,dt. \end{aligned}$$

Replacing *y* by $(\alpha x)/2$ and *ν* by $(2p+b-2)/4$, we obtain

$$\begin{aligned} \biggl( \int_{0}^{1}q(t) f(t)\,dt \biggr) \biggl( \int_{0}^{1}q(t) g(t)\,dt \biggr) = 2^{2p+b-4} \pi (\alpha x)^{1-p-\frac{b}{2}} \biggl( \Gamma \biggl( \frac{2p+b}{4} \biggr) \biggr) ^{2} \mathtt{J}^{2}_{ \frac{2p+b-2}{4}} \biggl( \frac{\alpha x}{2} \biggr) . \end{aligned}$$

Since *f* and *g* both are increasing on $(0, 1)$, it is evident from (23) that

$$\begin{aligned} {}_{2}\mathtt{B}_{b, p, \alpha^{2}}(x) & =\frac{x^{p}}{2^{2p+ \frac{b-3}{2}} { \Gamma { ( \frac{2p+b-1}{4} ) }} { \Gamma { ( \frac{2p+b+1}{4} ) }}} \biggl( \int_{0} ^{1} \bigl(1-t^{2} \bigr)^{\frac{2p+b-5}{4}} \cos \biggl( \frac{\alpha xt}{2} \biggr)\,dt \biggr) \\ & \quad {} \times \biggl( \int_{0}^{1} \bigl(1-t^{2} \bigr)^{\frac{2p+b-3}{4}} \cos \biggl( \frac{ \alpha xt}{2} \biggr)\,dt \biggr) \\ &=\frac{x^{p}}{2^{2p+\frac{b-3}{2}} { \Gamma { ( \frac{2p+b-1}{4} ) }} { \Gamma { ( \frac{2p+b+1}{4} ) }}} \biggl( \int_{0} ^{1}p(t)f(t) g(t)\,dt \biggr) \biggl( \int_{0}^{1}p(t)\,dt \biggr) \\ &\geq \frac{ \pi \alpha^{1-p-\frac{b}{2}}x^{1-\frac{b}{2}} ( \Gamma ( \frac{2p+b}{4} ) ) ^{2}}{2^{\frac{5-b}{2}} \Gamma ( \frac{2p+b-1}{4})\Gamma (\frac{2p+b+1}{4})}\; \mathtt{J}^{2}_{ \frac{2p+b-2}{4}} \biggl( \frac{\alpha x}{2} \biggr) . \end{aligned}$$

The subordinant for ${}_{2}\mathtt{B}_{b, p, - \alpha^{2}}$ is readily established in a similar manner by choosing

$$ q(t) = \bigl(1-t^{2} \bigr)^{\frac{2p+b-3}{4}} \cosh \biggl( \frac{\alpha x t}{2} \biggr) ; \quad \quad f(t)= g(t) := \bigl(1-t^{2} \bigr)^{-\frac{1}{4}}, \quad 0 < t < 1. $$

 □

### Remark 8

As a final application, choose $b=3$ and $\alpha =1$ in Theorem 6. Then

$$ {}_{2}\mathtt{B}_{3, p, 1}(x) \geq \frac{ \pi x^{-\frac{1}{2}} ( \Gamma ( \frac{2p+3}{4} ) ) ^{2}}{2 \Gamma (\frac{2p+2}{4}) \Gamma (\frac{2p+4}{4})}\; \biggl( \mathtt{J}_{\frac{2p+1}{4}} \biggl( \frac{ x}{2} \biggr) \biggr) ^{2}. $$

Remark 1 now shows that

$$ J_{\frac{p}{2}} \biggl( \frac{x}{ 2} \biggr) J_{\frac{p+1}{2}} \biggl( \frac{x}{ 2} \biggr) \geq \frac{ \sqrt{\pi } ( \Gamma ( \frac{2p+3}{4} ) ) ^{2}}{2 \Gamma (\frac{2p+2}{4})\Gamma (\frac{2p+4}{4})}\; \biggl( \mathtt{J} _{\frac{2p+1}{4}} \biggl( \frac{ x}{2} \biggr) \biggr) ^{2}. $$

Similarly,

$$ {}_{2}\mathtt{B}_{3, p, -1}(x) \geq \frac{ \pi x^{-\frac{1}{2}} ( \Gamma ( \frac{2p+3}{4} ) ) ^{2}}{2 \Gamma ( \frac{2p+2}{4})\Gamma (\frac{2p+4}{4})}\; \biggl( \mathtt{I}_{ \frac{2p+1}{4}} \biggl( \frac{ x}{2} \biggr) \biggr) ^{2}, $$

and thus

$$ I_{\frac{p}{2}} \biggl( \frac{x}{ 2} \biggr) I_{\frac{p+1}{2}} \biggl( \frac{x}{ 2} \biggr) \geq \frac{ \sqrt{\pi } ( \Gamma ( \frac{2p+3}{4} ) ) ^{2}}{2 \Gamma (\frac{2p+2}{4})\Gamma (\frac{2p+4}{4})}\; \biggl( \mathtt{I} _{\frac{2p+1}{4}} \biggl( \frac{ x}{2} \biggr) \biggr) ^{2}. $$

## Concluding remarks

This paper derived representation relations and functional inequalities for the extended Bessel function

$$ {}_{a}\mathtt{B}_{b, p, c}(x):= \sum _{k=0}^{\infty }\frac{(-c)^{k}}{k! \Gamma { ( a k +p+\frac{b+1}{2} ) } } \biggl( \frac{x}{2} \biggr) ^{2k+p} $$

in terms of the parameters *a*, *b*, and *p*. An important consequence is the $(a+1)$-order differential equation satisfied by the function ${}_{a}\mathtt{B}_{b,p, c}$. Monotonicity properties of ${}_{a} \mathtt{B}_{b,p, c}$ are obtained for non-positive *c*. Connections with earlier works on the Bessel function and its generalizations are also made.

Additionally, this paper also studied log-concavity and log-convexity properties in terms of the parameters *d* and *p* for the closely related function

$$ {}_{a}\mathcal{B}^{d}_{b,p, c}(x): =\sum _{k=0}^{\infty }\frac{(-c/4)^{k}\Gamma { ( p+\frac{b+1}{2} ) }}{ \Gamma { ( k+1 ) } \Gamma { ( ak+p+ \frac{b+1}{2} ) }} \frac{(d)_{k}}{k!}x^{k}. $$

As a consequence, direct and reverse Turán-type inequalities are obtained.
